# Supplementary material for: East Asian summer rainfall stimulated by subseasonal Indian monsoonal heating
Source: Nat Commun. 2023 Sep 22;14:5932. doi: 10.1038/s41467-023-41644-5 (PMC10517143; doi:10.1038/s41467-023-41644-5)
Supplement: Supplementary file 1 — Supplementary Information [file 41467_2023_41644_MOESM1_ESM.pdf]

Supporting Information for

East Asian summer rainfall stimulated by subseasonal Indian monsoonal heating

Shixue Li[1]*, Tomonori Sato[2], Tetsu Nakamura[2,3], Wenkai Guo[4]

[1]Graduate School of Environmental Science, Hokkaido University, Sapporo 060-0810, Japan

[2]Faculty of Environmental Earth Science, Hokkaido University, Sapporo 060-0810, Japan

[3]Climate Prediction Division, Japan Meteorological Agency, Tokyo 105-8431, Japan

[4]Faculty of Geosciences and Environmental Engineering, Southwest Jiaotong University, Chengdu 611756, China

Contents of this file

Supplementary Figures 1–16

Introduction

This is the supporting information containing figures referred to in the main article.

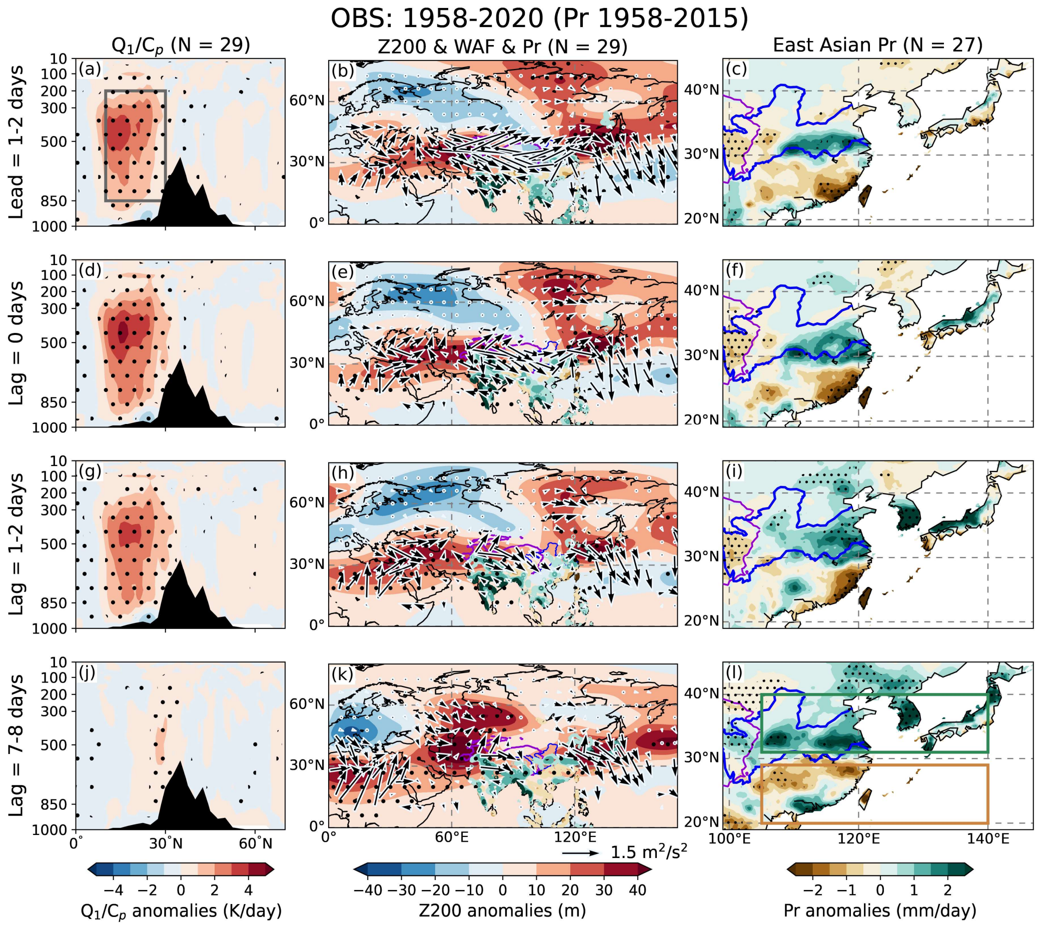

Supplementary Fig. 1 As in Figure 1 of the main article, but for the observations. The green and brown rectangles in (l) indicate the wet and dry band areas, respectively.

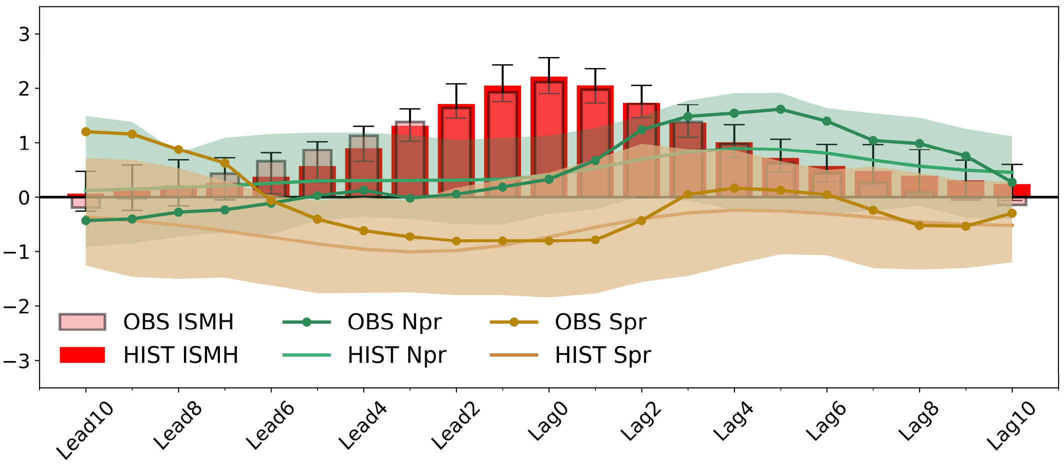

Supplementary Fig. 2 Lead–lag composite time series of diabatic heating anomalies (unit: K/day) averaged over 10°–30°N, 70°–85°E and vertical mass-weighted from 850 to 200 hPa (ISMH) and area-averaged precipitation anomalies (unit: mm/day) for wet band area (Npr) and the dry band area (Spr). The areas for Npr and Spr are indicated in Supplementary Figure 1. The error bars and shading represent the 100–member ensemble range obtained from large ensemble historical climate experiment (HIST).

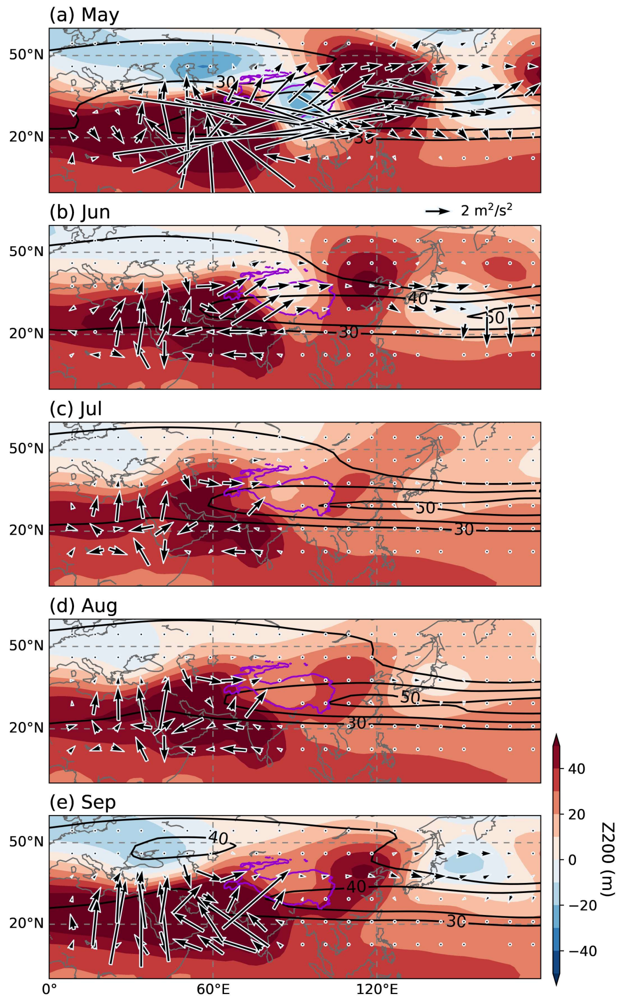

Supplementary Fig. 3 The responses of the 200-hPa geopotential height (shading) and the wave activity flux (arrows) to the Indian monsoon diabatic heating simulated by linear baroclinic model (LBM). Results are averaged from 30 to 50 days when LBM reached equilibrium. (a) experiment using May basic state, (b–e) as in (a) but for June to September basic states, respectively. The contours represent the 200-hPa zonal wind (unit: m/s) from basic states.

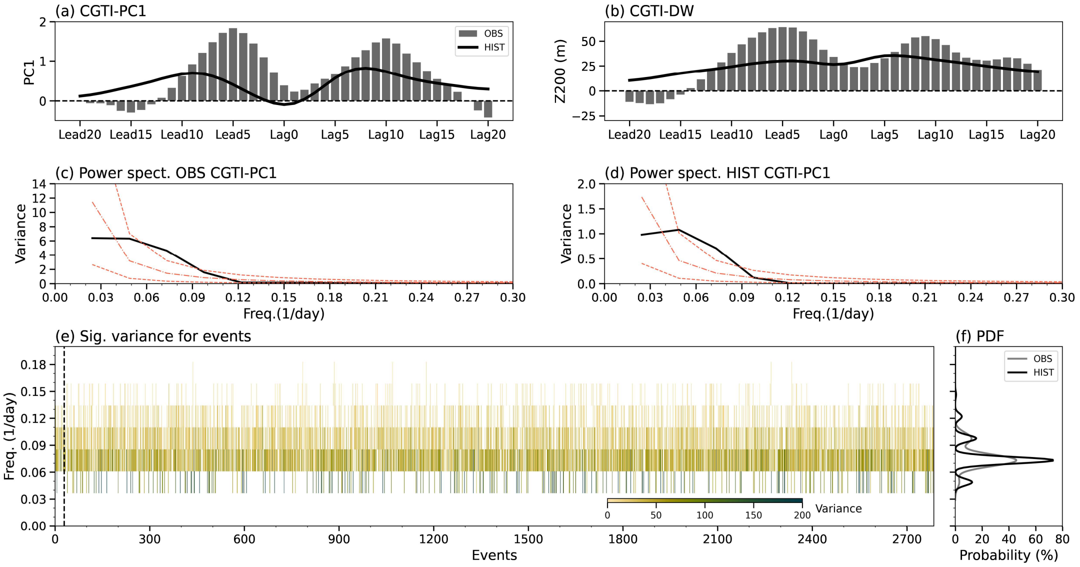

Supplementary Fig. 4 Time variation of the circumglobal teleconnection (CGT) from lead of 20 days to lag of 20 days represented by (a) CGT index based on PC1 of June to September interannual 200-hPa meridional wind (CGTI–PC1; Supplementary Fig. 11) and (b) CGT index based on 200-hPa geopotential height averaged over 35°–40°N, 60°–70°E (CGTI–DW). Power spectrum analysis of (c) CGTI–PC1 of observation and (d) CGTI–PC1 of large ensemble historical climate experiment (HIST), respectively, where the dash-dot and dashed red curve denote the red noise and its 95% significance boundary. (e) A list of the spectral variance values for each event of CGTI–PC1. The range of significant frequency (day^{-1}) exceeding the 95% red noise confidence interval from the power spectrum are plotted, and the color shading represents the spectral variance. The vertical dash line is for the demarcation of the events collected from observation (left) and HIST (right). (f) probability distribution function (PDF) of the frequency based on (e).

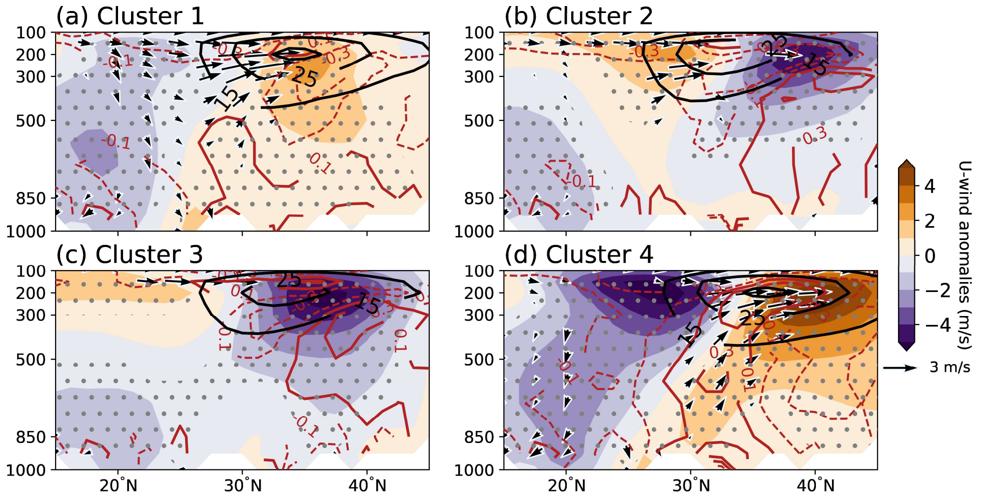

Supplementary Fig. 5 Cross section along 120°E showing the anomalous wind field based on large ensemble historical climate experiment (HIST) for (a) cluster 1, (b) cluster 2, (c) cluster 3, and (d) cluster 4 at event0 day. The color shading represents zonal wind anomalies, the arrows indicate anomalous meridional wind with vertical velocity, and the black and red contours depict reconstructed zonal wind velocity (unit: m/s) corresponding jet patterns and temperature advection anomalies (unit: K/day), respectively. Stippled regions and arrows indicate anomalies that are statistically significant at the 99% confidence level based on the two-sided Student's t test.

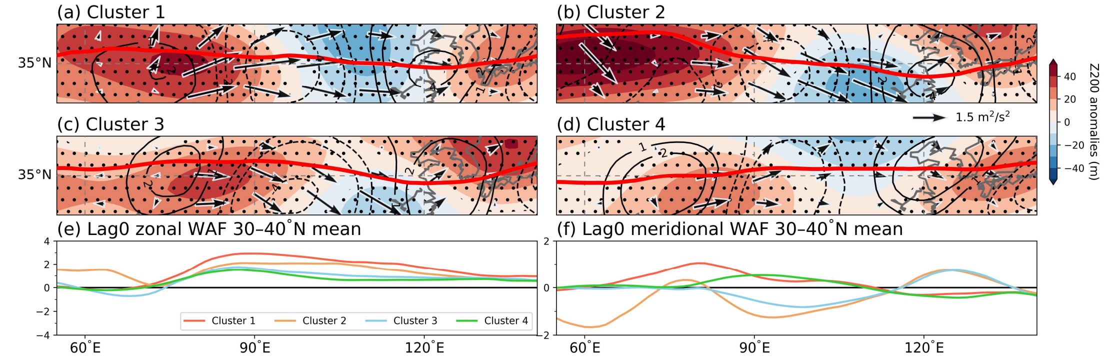

Supplementary Fig. 6 Composite based on large ensemble historical climate experiment (HIST) of jet axis at event0 day (thick red curve), 200-hPa geopotential height anomalies at lag of 0 days (shading), and 200-hPa meridional wind anomalies (contours; unit: m/s) together with wave activity flux at lag of 0 days (arrows). (a) cluster 1, (b) cluster 2, (c) cluster 3, and (d) cluster 4. (e) and (f) depict zonal and meridional component of the wave activity flux (unit: m^2/s^2) averaged over 30°–40°N at lag of 0 days, respectively, for each cluster. Stippled regions indicate anomalies that are statistically significant at the 99% confidence level based on the two-sided Student's t test.

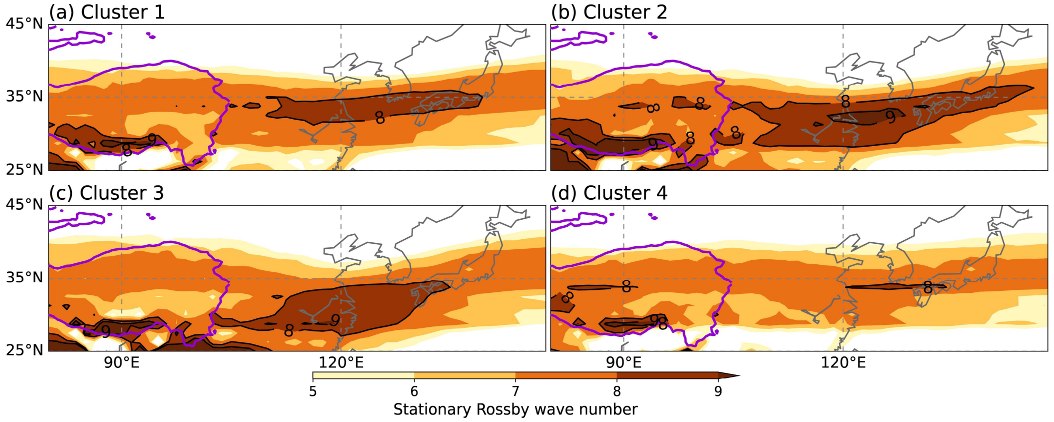

Supplementary Fig. 7 Stationary Rossby wavenumber for each cluster (a) cluster 1, (b) cluster 2, (c) cluster 3, and (d) cluster 4. The wavenumber is determined based on Figure 3 in the main article.

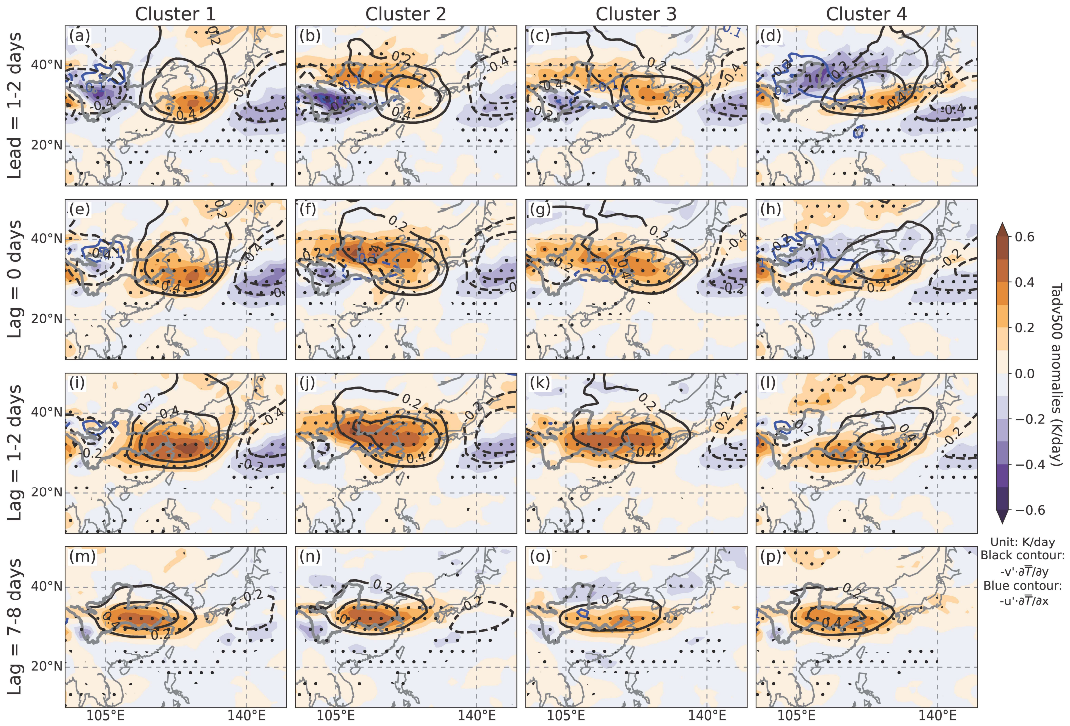

Supplementary Fig. 8 Evolution of 500-hPa temperature advection anomalies on (a–d) lead of 1–2 days, (e–h) lag of 0 days, (i–l) lag of 1–2 days, and (m–p) lag of 7–8 days based on large ensemble historical climate experiment (HIST). (a, e, i, m) cluster 1, (b, f, j, n) cluster 2, (c, g, k, o) cluster 3, and (d, h, l, p) cluster 4. The color shading represents the total 500-hPa temperature advection anomalies, and the black and blue contours indicate the anomalous 500-hPa temperature advections computed using anomalous meridional and zonal winds with climatological temperature gradient, respectively. Stippled regions indicate anomalies that are statistically significant at the 99% confidence level based on the two-sided Student's t test.

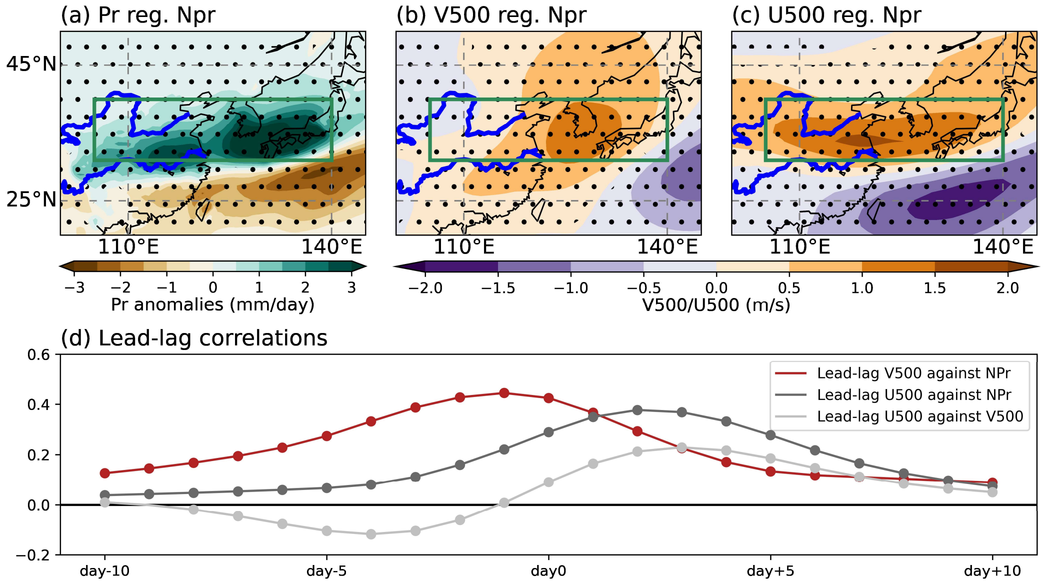

Supplementary Fig. 9 The spatial pattern of (a) precipitation, and 500-hPa (b) meridional wind (V500), and (c) zonal wind (U500) regressed upon standardized daily wet band precipitation (Npr) in June created using large ensemble historical climate experiment (HIST). The green box denotes the wet band area. Stippled regions indicate anomalies that are statistically significant at the 99% confidence level based on the two-sided Student's t test. (d) lead–lag correlations among Npr, V500, and U500 averaged over the wet band area in June. The V500 lead–lag Npr means correlation of lead–lagged 500-hPa meridional wind against Npr, and similar manner for others. Lines with solid circle denotes correlations satisfy two-sided p<0.01.

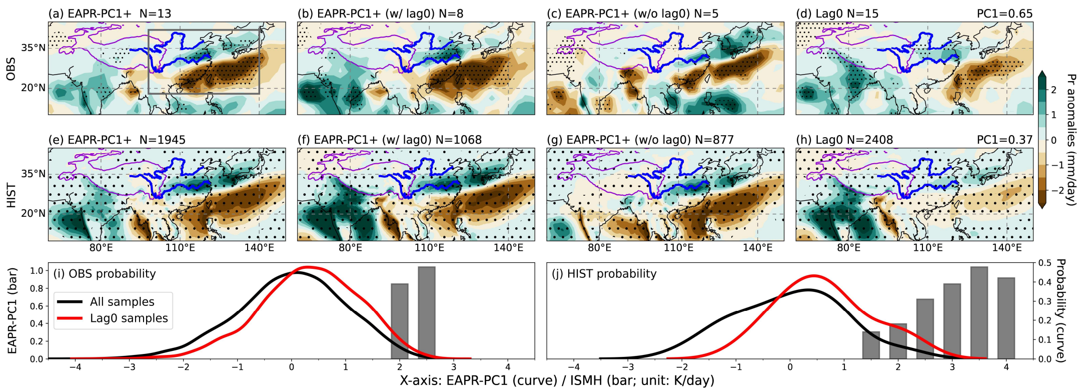

Supplementary Fig. 10 Composite June monthly mean precipitation pattern based on (a) years with first leading mode of June monthly mean East Asian precipitation (EAPR–PC1) greater than 0.5 (EAPR–PC1+), (b) years with both EAPR–PC1 greater than 0.5 and with detection of lag0, (c) years with EAPR–PC1 greater than 0.5 but without lag0, and (d) years with lag0 only. (i) probability (right y-axis) of the EAPR–PC1 (x-axis) distribution for all samples (black curve) and the samples with lag0 (red curve), where the grey bars represent the EAPR–PC1 score (left y-axis) estimated from the conditioned lag0 Indian monsoon heating (ISMH) magnitude for each 1 K/day interval (x-axis). (e–h) and (j) are similar as (a–d) and (i), respectively, but for large ensemble historical climate experiment (HIST). Dotted regions denote anomalies significant at the 90% and 99% confidence level for the observations and HIST, respectively. Number of samples used for the composite analysis are indicated as N. The projected EAPR–PC1 values are shown on the top right of (d) and (h). The domain to obtain EAPR–PC1 is shown in the grey box in (a). The ISMH is averaged over 10°–30°N, 70°–85°E and applied vertical mass-weighted from 850 to 200 hPa.

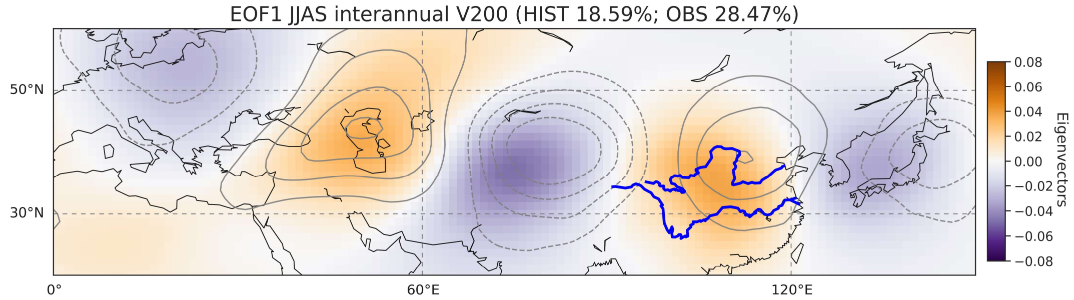

Supplementary Fig. 11 The EOF1 eigenvectors of interannual June to September seasonal mean meridional wind anomalies at 200-hPa. The contour is the result for JRA-55 reanalysis (interval: 0.01) and the color shading is for the result of large ensemble historical climate experiment (HIST).

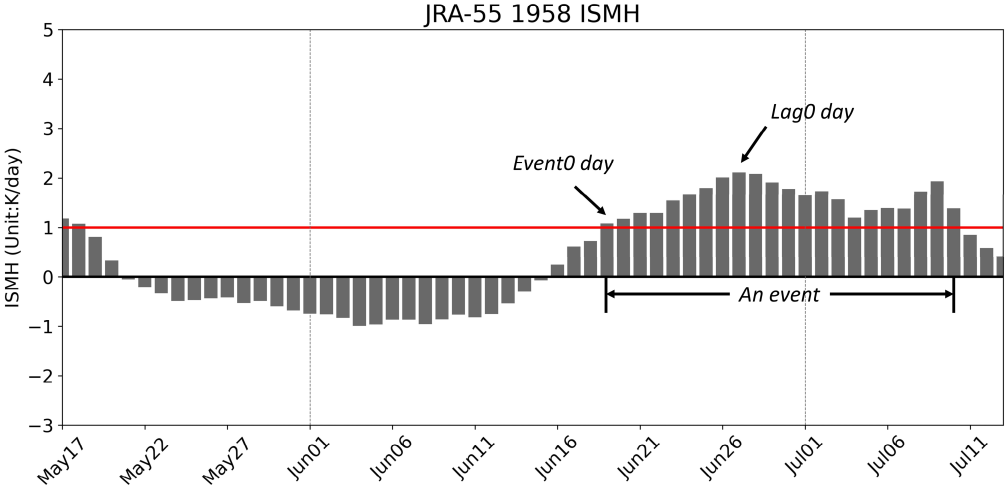

Supplementary Fig. 12 Schematic illustrating the method adopted for identification of strong diabatic heating over the Indian summer monsoon region. Bars are diabatic heating from 17 May 1958 to 13 July 1958 based on JRA-55, averaged over 10°–30°N, 70°–85°E and vertically mass-weighted from 850 to 200 hPa. Red line is the threshold (i.e., 1 K/day).

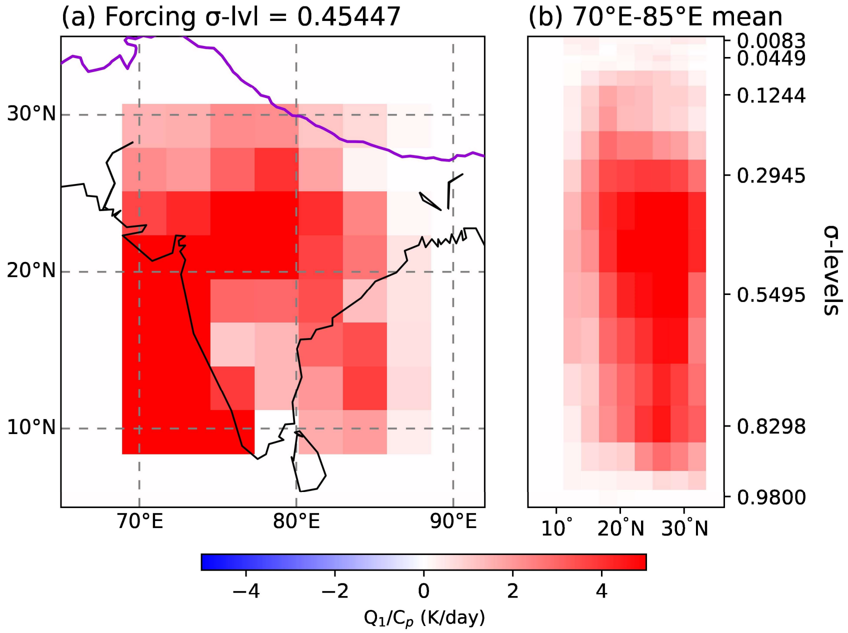

Supplementary Fig. 13 The diabatic heating pattern that was used as forcing in linear baroclinic model.

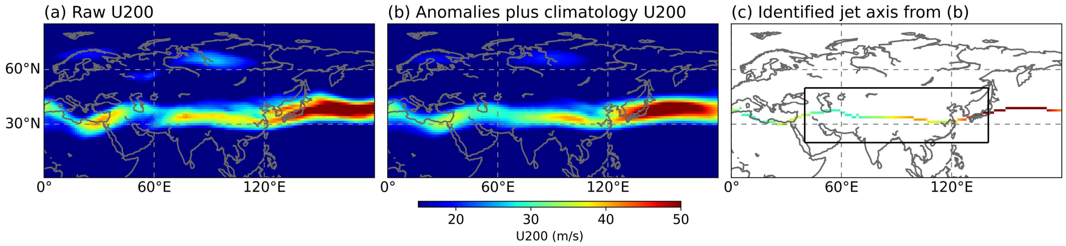

Supplementary Fig. 14 Schematic showing an example of 200-hPa zonal wind based on (a) original data, (b) reconstructed data, and (c) identification of the jet axis from (b).

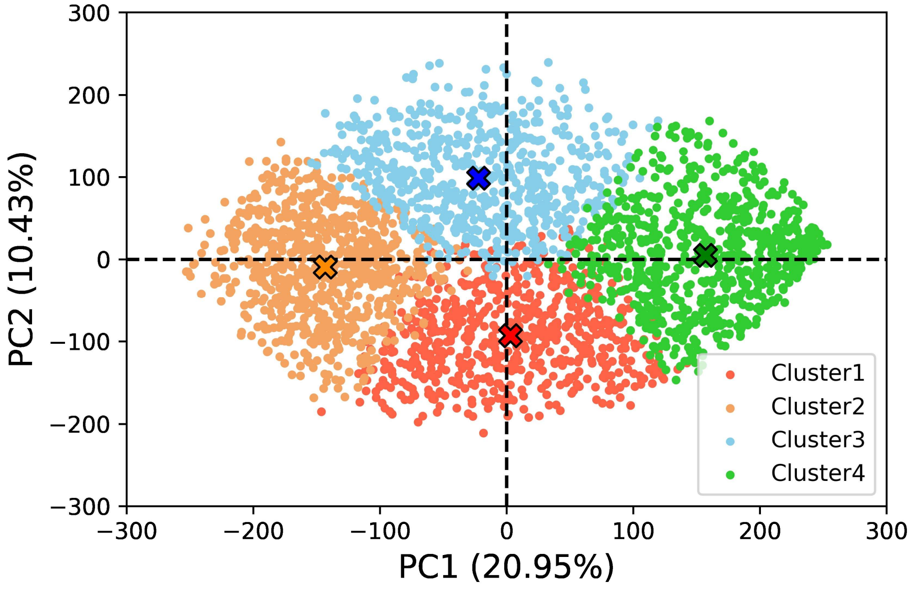

Supplementary Fig. 15 Comparison of four clusters about the jet pattern created by K-means clustering and two leading modes from principal component analysis. The percentage values mean the proportion of explanation for PC1 and PC2. The crosses represent the group mean of each cluster.

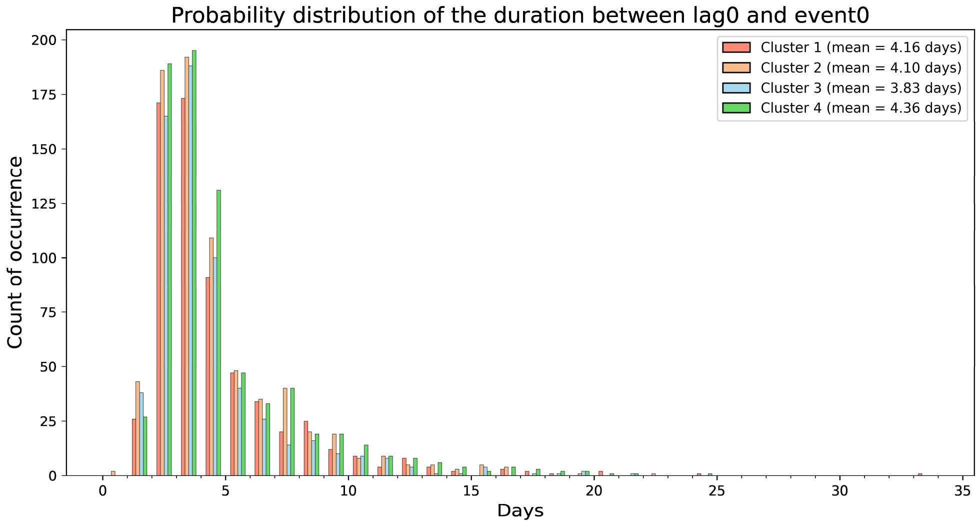

Supplementary Fig. 16 Probability distribution of the number of days between event0 day and lag0 day in each cluster.
